# Spatial heterogeneity of prion gene polymorphisms in an area recently infected by chronic wasting disease

**DOI:** 10.1080/19336896.2019.1583042

**Published:** 2019-03-19

**Authors:** William L. Miller, W. David Walter

**Affiliations:** a Pennsylvania Cooperative Fish and Wildlife Research Unit, Department of Ecosystem Science and Management, Intercollege Graduate Degree Program in Ecology, The Pennsylvania State University, University Park, PA, USA; b U.S. Geological Survey, Pennsylvania Cooperative Fish and Wildlife Research Unit, The Pennsylvania State University, University Park, PA, USA

**Keywords:** Prion protein gene polymorphism, spatial variability, white-tailed deer, chronic wasting disease, sequence analysis

## Abstract

Genetic variability in the prion protein (*Prnp*) gene influences host susceptibility to many pathogenic prion diseases. Understanding the distribution of susceptible *Prnp* variants and determining factors influencing spatial genetic patterns are important components of many chronic wasting disease mitigation strategies. Here, we describe *Prnp* variability in white-tailed deer (*Odocoileus virginianus*) from the Mid-Atlantic region of the United States of America, an area with a recent history of infection and low disease incidence. This population is characterized by lower rates of polymorphism and significantly higher frequencies of the more susceptible 96GG genotype compared to previously surveyed populations. The prevalence of the most susceptible genotypes at disease-associated loci did vary among subregions, indicating that populations have innate differences in genotype-dictated susceptibility.

## Introduction

Infectious diseases are important population stressors that can have significant impacts on the health of natural populations as well as their demographic and genetic trajectories [1,2]. Emergent diseases or novel outbreaks in naïve populations can act indiscriminately, with all individuals being similarly susceptible to transmission [3]. The dynamics of many transmissible diseases, however, can be influenced by genetic variability, which makes certain populations less susceptible to infection than others [4]. In these cases, the occurrence and frequency of disease-associated alleles can shape the emergence, progression, and outcome of outbreaks [5,6]. Existing genetic diversity at disease-associated loci may be especially important in determining the trajectory of recently emerging pathogens. Understanding differential patterns of host susceptibility can aid in the development of mitigation strategies that are targeted towards those populations that are most vulnerable and susceptible [7].

Genetic variation in the prion protein (*Prnp*) gene influences susceptibility to transmissible spongiform encephalopathies [8–10]. These diseases are a family of fatal neurodegenerative diseases caused by an infectious, misfolded isoform of the prion protein [11]. Transmissible prion diseases affect many mammalian species, including humans, sheep, and cervids [11]. Direct contact, and in particular, the ingestion of infected material or tissues, seems to be the primary mode of transmission for most acquired prion diseases [12]. Other studies, however, support environmental and fluid-mediated transmission of certain prion diseases, such as scrapie and chronic wasting disease [13,14]. Many outbreaks of transmissible spongiform encephalopathies seem to affect or originate from agriculturally-propagated species, such as sheep (*Ovis aries*) and cows (*Bos taurus*) [15]. One notable exception is chronic wasting disease, which has affected free-ranging cervid populations in North America and, most recently, Scandinavia [16,17]. These species are the subject of considerable management interest given their ecological, cultural, and economic importance as a game species. Thus, there is a strong impetus to understand factors dictating susceptibility to chronic wasting disease, which has become a widespread concern given its negative effects on free-ranging populations [18].

Nonsynonymous polymorphisms in the *Prnp* gene have been consistently linked to differences in relative susceptibility for many species affected by prion diseases [10,19,20]. Although these polymorphisms rarely provide complete protection from prion diseases, they often lead to substantial reductions in genotype-specific prevalence rates. For example, Robinson et al. [21] observed a four-fold reduction in the prevalence and infection rates of chronic wasting disease among white-tailed deer (*Odocoileus virginianus*) with a rare serine substitution at codon 96 compared to those deer that were homozygous for the wild-type protein (glycine at codon 96). Similar polymorphisms have been observed to affect susceptibility in other species where infection has occurred [22]. Understanding the occurrence and distribution of these rare variants within free-ranging cervid populations can provide insights into the potential epizootiology of chronic wasting disease outbreaks. Because the force of infection differs significantly among genotypes [21], prevalence rates may tend to be lower in populations with a higher frequency of less susceptible genotypes. Additionally, epidemiological models predict the long-term stabilization of population dynamics when incorporating genotypic differences in susceptibility and infectivity, which contrasts a pattern of continual decline when not accounted for [23]. Thus, genetic variability in the *Prnp* gene likely influences the trajectory and outcome of disease outbreaks and likely influences predictions on population-level responses.

Genetic variation is rarely homogeneous across landscapes. This is a trend that also seems to be true of *Prnp* variability among cervid populations [24–28]. Measuring the spatial variation of *Prnp* genotypes may prove useful in understanding the relative susceptibility, progression, and outcome of chronic wasting disease epidemics in relation to other populations. Previous studies have largely been non-spatial and have focused on comparing patterns of *Prnp* variation among infected and uninfected individuals or characterizing the genotype frequencies of a single region [21,28,29]. This may not provide accurate estimates of the innate spatial heterogeneity in genotype frequencies across broader landscapes. Initial heterogeneity can be substantial among populations unaffected by chronic wasting disease [30], so understanding patterns of local *Prnp* variation is important for identifying local populations that may be more susceptible to disease establishment. Given that chronic wasting disease outbreaks tend to be locally constrained, at least initially [31], measuring spatial patterns of *Prnp* variability may also prove more useful in characterizing and predicting local selection dynamics. Thus, a more thorough evaluation of levels of spatial heterogeneity is desirable, especially in regions where the disease is constrained in distribution and where prevalence rates within and among populations are low.

### Objectives

**Figure 1.**
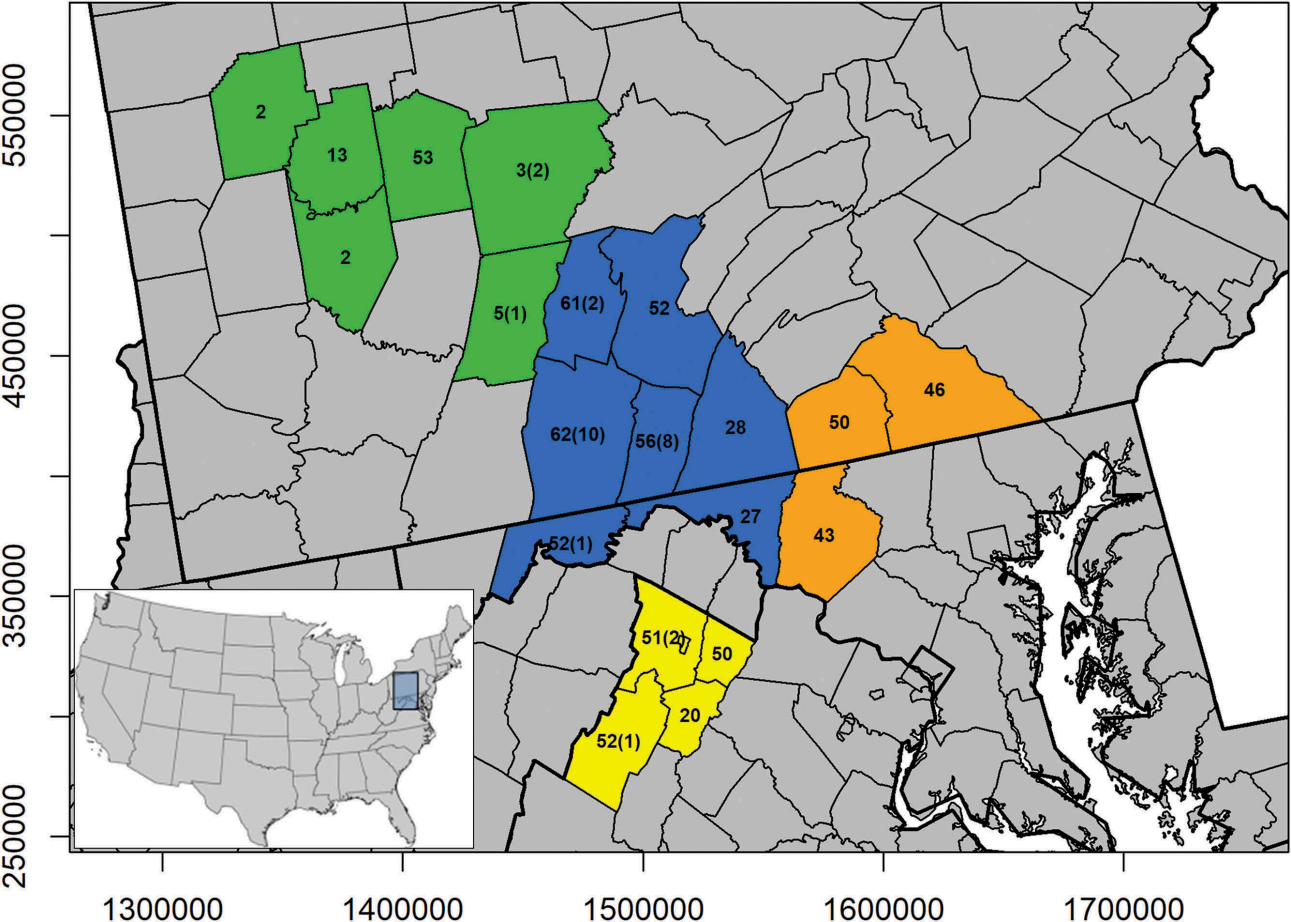
The distribution and sample size of white-tailed deer used to determine *Prnp* variability in the Mid-Atlantic region. Samples were georeferenced by sampling unit (county). The number of infected samples genotyped are indicated in parentheses. Sampling units were stratified into four subregions (orange = one, blue = two, green = three, and yellow = four) that generally conformed to predicted dispersal barriers and disease management units.

Here, we describe underlying frequencies of *Prnp* genotypes in an area of relatively recent infection in the Mid-Atlantic region of United States of America. We specifically focused on determining patterns of population-level susceptibility, defined as the frequency of the most susceptible *Prnp* genotypes, for the main species impacted by chronic wasting disease in the region (white-tailed deer). Levels of susceptibility were compared to studies conducted in regions that have a longer history of chronic wasting disease in order to determine the relative susceptibility of this region to emerging infection. Genotype frequencies were then compared among four subregions and 20 sampling localities to assess how geographic patterns of susceptibility may change across landscapes (Figure 1).

## Results

A total of 701 uninfected deer and 27 infected deer were successfully sequenced from white-tailed deer populations in the Mid-Atlantic region of the United States. These areas are characterized by low prevalence rates (<1% in subregions 2 and 4) [32,33] or no disease incidence (subregion 1) in free-ranging populations. Chronic wasting disease was first detected in subregion 3 in 2015 along the border with subregion 2. Since then, only three additional cases have been detected in subregion 3. Prevalence estimates are currently unavailable for this region, but the low number of detected cases indicates that prevalence is likely less than subregions 2 and 4. While we were unable to receive samples from all deer that tested positive for chronic wasting disease in this region but the 27 reflected a representative sample across the region (Appendix A).

We detected nucleotide variability at 12 single nucleotide polymorphism loci within the open-reading frame of the *Prnp* gene (Table 1). Two novel polymorphisms were observed (nucleotides 110 and 499), but were rare (frequency < 1.0%). Five polymorphisms were nonsynonymous, including a novel polymorphism (codon 37: G→V substitution) and previously documented substitutions at codons 123 (A→T substitution) and 226 (Q→K substitution). The remaining two nonsynonymous polymorphisms occurred at loci previously linked to chronic wasting disease susceptibility (codon 95: Q→H substitution, codon 96: G→S substitution) [25,26,28,34]. The most susceptible allele was in highest frequency for both sites (95Q = 95.2%; 96G = 91.8%). Locus 347 (codon 116) was fixed for the most susceptible allele. Population-wide genotype frequencies deviated from Hardy-Weinberg expectations for both disease-associated loci, but only codon 95 deviated from Hardy-Weinberg expectations in one sampling location after accounting for potential substructure.

**Table 1.** The frequency and count (in parentheses) of *Prnp* genotypes for white-tailed deer sampled from four subregions in the Mid-Atlantic region of the United States. Loci are designated by nucleotide. Codons are listed in parentheses for nonsynonymous loci. Crosses (†) indicate loci found to be associated with chronic wasting disease susceptibility in previous studies.

Locus	Genotype	Amino Acid	1	2	3	4
60	CC		0.696 (96)	0.834 (282)	0.846 (66)	0.884 (153)
	CT		0.239 (33)	0.115 (39)	0.115 (9)	0.104 (18)
	TT		0.065 (9)	0.050 (17)	0.038 (3)	0.012 (2)
110 (37)	GG	GG	0.986 (137)	1.000 (338)	1.000 (78)	1.000 (172)
	GT	GV	0.014 (2)	0.000 (0)	0.000 (0)	0.000 (0)
	TT	VV	0.000 (0)	0.000 (0)	0.000 (0)	0.000 (0)
153	CC		0.633 (88)	0.728 (246)	0.936 (73)	0.780 (135)
	CT		0.237 (33)	0.189 (64)	0.064 (5)	0.179 (31)
	TT		0.129 (18)	0.083 (28)	0.000 (0)	0.040 (7)
285 (95)†	AA	QQ	0.878 (122)	0.902 (305)	0.949 (74)	0.960 (166)
	AC	QH	0.100 (14)	0.080 (27)	0.051 (4)	0.040 (7)
	CC	HH	0.021 (3)	0.018 (6)	0.000 (0)	0.000 (0)
286 (96)†	GG	GG	0.892 (124)	0.885 (299)	0.679 (53)	0.884 (153)
	GA	GS	0.072 (10)	0.098 (33)	0.231 (18)	0.098 (17)
	AA	SS	0.036 (5)	0.018 (6)	0.090 (7)	0.017 (3)
324	AA		0.986 (137)	0.982 (332)	0.949 (74)	0.994 (172)
	AG		0.014 (2)	0.018 (6)	0.051 (4)	0.000 (0)
	GG		0.000 (0)	0.000 (0)	0.000 (0)	0.006 (1)
367 (123)	GG	AA	1.000 (139)	1.000 (338)	0.987 (77)	1.000 (173)
	GA	AT	0.000 (0)	0.000 (0)	0.013 (1)	0.000 (0)
	AA	TT	0.000 (0)	0.000 (0)	0.000 (0)	0.000 (0)
378	GG		1.000 (139)	0.994 (336)	1.000 (78)	1.000 (173)
	GA		0.000 (0)	0.006 (2)	0.000 (0)	0.000 (0)
	AA		0.000 (0)	0.000 (0)	0.000 (0)	0.000 (0)
438	CC		0.799 (111)	0.893 (302)	0.949 (74)	0.624 (108)
	CT		0.151 (21)	0.083 (28)	0.051 (4)	0.306 (53)
	TT		0.050 (7)	0.024 (8)	0.000 (0)	0.069 (12)
499	AA		1.000 (139)	1.000 (338)	1.000 (78)	0.988 (171)
	AC		0.000 (0)	0.000 (0)	0.000 (0)	0.012 (2)
	CC		0.000 (0)	0.000 (0)	0.000 (0)	0.000 (0)
555	CC		0.676 (94)	0.547 (185)	0.449 (35)	0.514 (89)
	CT		0.216 (30)	0.322 (109)	0.295 (23)	0.370 (64)
	TT		0.108 (15)	0.130 (44)	0.256 (20)	0.116 (20)
676 (226)	CC	QQ	0.993 (137)	0.997 (337)	0.974 (76)	0.983 (169)
	CA	QK	0.007 (1)	0.003 (1)	0.000 (0)	0.006 (1)
	AA	KK	0.000 (0)	0.000 (0)	0.026 (2)	0.116 (2)

**Figure 2.**
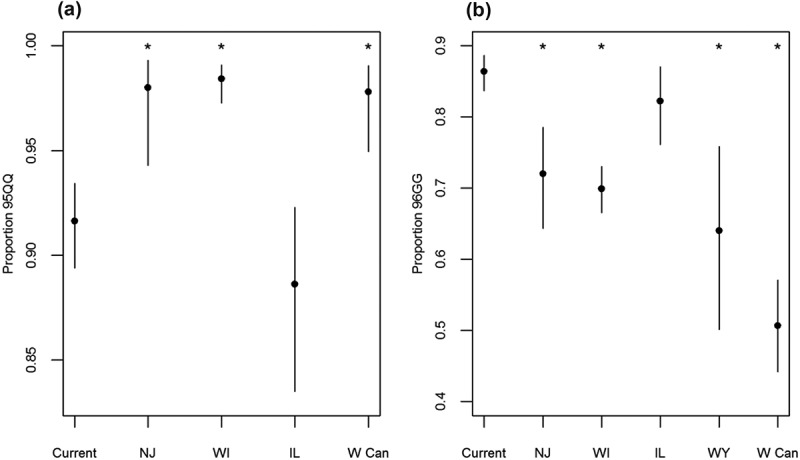
Comparison of the genotype frequencies for (a) codon 95 and (b) codon 96 among North American white-tailed deer populations (Current = Current study region; NJ = New Jersey; WI = Wisconsin; IL = Illinois; WY = Wyoming; W Can = western Canada). Asterisks indicate genotype frequencies that are significantly different from those in the study region, as determined by Fisher’s exact tests. Bars indicate 95% confidence intervals for genotype frequencies.

Genotype frequencies observed in this study were compared to other regions in order to assess differences among distinct areas within the range of white-tailed deer. The proportion of the most susceptible genotype at codon 95 (95QQ) was 91.6% for the Mid-Atlantic region and was significantly lower than the frequencies of this genotype in populations from New Jersey (98.0%, *F* = 0.223, *P’* = 0.0497) [35], Wisconsin (98.4%, *F* = 0.175, *P’* < 0.0001) [25,28], and western Canada (97.8%, *F* = 0.247, *P’* = 0.0080; Figure 2) [29]. The proportion of the most susceptible genotype at codon 96 (96GG) was 86.4% and was significantly higher than the frequencies of this genotype in populations from New Jersey (72.0%, *F* = 2.468, *P’* = 0.0006) [35], Wisconsin (69.9%, *F* = 2.733, *P’* < 0.0001) [25,28], Wyoming (64.0%, *F* = 3.566, *P’* = 0.0018) [36], and western Canada (50.7%, *F* = 6.172, *P’* < 0.0001; Figure 2) [29]. The degree of deviance observed was substantial, with the frequency of the 96GG genotype differing, on average, by 22.3% and by as much as 35.7%. Observed genotype frequencies were not significantly different from Illinois populations, although the frequency of the most susceptible genotypes was lower for Illinois in both cases (88.6% and 82.3% for codons 95 and 96, respectively; Figure 2) [26]. Most infected deer expressed the most susceptible genotypes at both codons with the exception of two deer that were heterozygous for codon 96 (96GS genotype; Appendix A).

**Figure 3.**
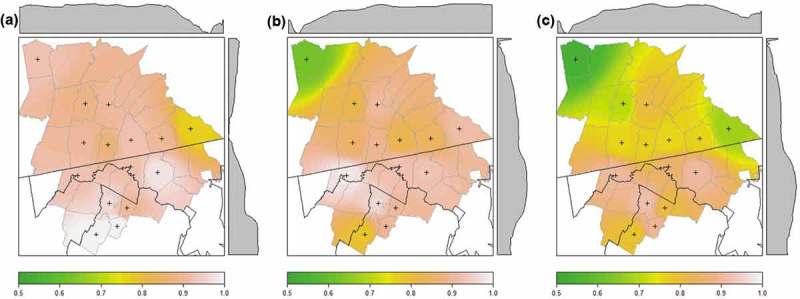
Surfaces showing the interpolated frequencies of the most susceptible genotypes at (a) codon 95, (b) codon 96, and (c) joint genotypes for white-tailed deer in the Mid-Atlantic region of the United States. Axis plots display spatial trends in the frequency of the most susceptible genotype. Sampling units used for interpolation are marked with a cross (N ≥ 20 individuals).

The frequencies of the most susceptible genotypes varied among sampling regions (95QQ = 87.8% to 96.0%; 96GG = 68.0% to 89.2%; Table 1). Frequencies of the 95QQ genotype did not differ significantly among subregions, although subregion one was characterized by lower frequencies of this genotype (Table 1). This trend was driven by genotype frequencies within the eastern-most county, which deviated from the average frequency of the 95QQ genotype by 15.7% (Figure 3). Subregion three had a significantly lower frequency of 96GG individuals relative to all other subregions (subregion one: *F* = 0.258, *P’* = 0.0012; subregion two: *F* = 0.278, *P’* = 0.0002; subregion four: *F* = 0.279, *P’* = 0.0010). These differences were at least 20% lower than those observed in other regions (Table 1). Spatial interpolation plots show that sampling units within subregions tend to have similar genotype frequencies (Figure 3). There is a general trend towards increased susceptibility in south-central units for both codons. The lowest frequencies of susceptible genotypes at codon 95 occur in the south-eastern part of the study area, while susceptibility, as determined by the frequency of codon 96GG genotypes, increases in a northwest-to-southeast direction.

Inferred patterns of susceptibility decrease synergistically when both loci are considered jointly. The most common multi-locus genotype was the QG/QG genotype (78.2%; n = 569). Only one individual exhibited polymorphisms at both loci (QG/HS genotype), indicating the rarity of individuals with less susceptible alleles at multiple loci. The proportion of individuals that were doubly homozygous ranged from 62.8% (n = 49) in subregion three to 84.4% (n = 146) in subregion four. Spatial interpolation still exhibited a northwest-to-southeast cline in patterns of susceptibility, although the proportion of susceptible individuals was generally lower within sampling units when loci were considered jointly (Figure 3).

## Discussion

The observed variability within the *Prnp* gene was less in the Mid-Atlantic region than reported in previous studies. We observed five non-silent and seven silent polymorphisms. For comparison, populations from Midwestern states and western Canada reported 14 and 15 polymorphisms, respectively [26,29]. Many of the previously reported variants were quite rare (<5.0% frequency), so it may be possible that undetected variation exists. The total number of deer sampled in this study does match or exceed the sample size of previous studies, however. Additionally, few previously reported polymorphisms were in the first and last 100 bases of the amplified sequence where there is a higher density of lower quality calls (Phred < 20). This makes it unlikely that bases were missed due to sequence quality. These results may indicate lower *Prnp* variation within this population when compared to other regions.

We did detect novel polymorphisms at nucleotide positions 110 and 499. The change at position 110 led to a change in the amino acid sequence. This novel variant was identified in two individuals from subregion one. Given the frequency of this polymorphism within our sample and the fact that this polymorphism has not been observed in previous studies, we expect that the presence of this polymorphism in free-ranging populations is rare and is unlikely to contribute to susceptibility at the population-scale. Additionally, neither of these deer tested positive for chronic wasting disease, indicating that this polymorphism does not seem to cause a novel variant of the disease.

Variability was detected at two loci consistently linked to lower susceptibility to chronic wasting disease in previous studies [25,26,28,34]. Patterns of predicted susceptibility, as inferred from the frequencies of major allele homozygotes, were markedly different in this study region in comparison to others. We observed a significantly lower frequency of the 95QQ genotype relative to other study regions, with the exception of populations from Illinois (Figure 2). Despite this trend, the average deviation among study regions was only 6.8% and the frequency of the 95QQ genotype exceeded 88% in all cases (Figure 2). Thus, variation at this locus may influence individual susceptibility to chronic wasting disease but due to the rarity of genetic variants, codon 95 may have a limited influence on population-scale patterns of susceptibility. In contrast, the observed frequency of the 96GG genotype was higher than most previously described populations (Figure 2). This finding suggests that there may be an innate vulnerability to disease establishment in this population. Individual deer were rarely variable at both loci, which may have implications for individual health given that the protective benefits of genetic variability seem to be additive [26]. The fact that these loci do not covary may be beneficial for herd health, however, as it seems to increase the number of individuals with at least one less susceptible genotype relative to the frequencies of each locus individually.

Understanding the factors leading to *Prnp* variation is an important goal of many chronic wasting disease management strategies. One factor that has been suggested to drive underlying genetic variation is selection in favor of less susceptible genotypes [4,21]. Under this assumption, regions with higher prevalence rates are more likely to exhibit lower frequencies of susceptible genotypes over time due to differences in infection rates. Indeed, regions where chronic wasting disease has been present on the landscape for much longer time periods, such as Wyoming, exhibited significantly lower frequencies of the 96GG genotype relative to the Mid-Atlantic region (Figure 2) [36]. This indicates a shift towards the heterozygous and/or homozygous minor allele states in regions with higher disease prevalence, which are supposedly less susceptible to infection. Because chronic wasting disease is also characterized by a relatively patchy spatial distribution [31], selection may also play a role in dictating the local distribution and abundance of less susceptible alleles as well. Patterns of susceptibility within the Mid-Atlantic region, however, do not conform to the expected pattern of selection that is believed to drive genotype frequencies. The proportion of the 95QQ and 96GG genotypes was higher in regions with greater disease incidence (subregions two and four) and longest history of infection (2009 in the case of subregion four; Figure 3). Conversely, herds with the lowest frequencies of the most susceptible genotypes were found in areas with very limited disease incidence (subregion three) or no incidence (subregion one) in free-ranging deer (Figure 3). This is somewhat surprising, given that appreciable shifts in allele frequencies can be detected even when disease prevalence is low (< 1.0% prevalence) in other cervid populations (Rocky Mountain elk, *Cervus elaphus*) [4]. Because other studies did not evaluate fine-scale fluctuations in genotypic frequencies, it is difficult to determine if this is a trend specific to this region or if other regions show similar trends. Continued fine-scale genotyping efforts may aid in understanding factors that lead to differences in *Prnp* variability among closely-associated populations.

Demographic factors, such as dispersal and population structure, may also affect the spatial distribution of *Prnp* variability. This is a trend that has previously been postulated to affect the *Prnp* genotype frequencies of white-tailed deer and other cervids [21,26,30,37], and may influence the distribution of genotypes in this region. Region-wide genotype frequencies were diverged significantly from Hardy-Weinberg expectations, although the frequencies of these genotypes were not different from expected when samples were grouped by sampling unit. This finding suggests that stratification influences *Prnp* variability. Landscape features influence genetic connectivity in white-tailed deer and may explain regional variation in genotype frequencies [38]. This is a possible explanation for the differences in genotype frequencies observed between subregions, which are separated by large geophysical escarpments, large rivers, and/or high-volume traffic roads. It is currently unknown how these natural and anthropogenic barriers affect gene flow in this region. However, patterns of demographic dispersal are known to be influenced by such barriers in this region [39]. Thus, they are predicted to have similar impacts on gene flow and *Prnp* frequencies as well. Additionally, previous studies suggest that white-tailed deer also conform to a clinal pattern of genetic structure, which may be consistent with the north-to-south gradient of increasing genetic susceptibility observed in this study [40,41]. The magnitude of divergence in *Prnp* genotype frequencies was somewhat unexpected, especially given the widespread nature of white-tailed deer gene flow and relative permeability of previously reported dispersal barriers [38,42]. This further highlights the importance of understanding factors that give rise to initial patterns of *Prnp* heterogeneity. Estimating gene flow and population structure in this region using neutral genetic markers is an area of ongoing research and is likely elucidate the trends observed here.

### Conclusions

Attempts to manage chronic wasting disease in areas of recent emergence require an understanding of the factors influencing the susceptibility of populations. Here, we have reported lower *Prnp* variability at disease-associated loci than previously documented for white-tailed deer populations infected with chronic wasting disease. Genotype frequencies varied among subregions and even among counties within subregions. Therefore, spatial variability in *Prnp* genotype frequencies seems to occur at finer scales than previously reported. Focusing on describing genotype frequencies at a regional-scale may under- or over-represent the genotype frequencies of disease-associated loci within distinct subpopulations. These results highlight the importance of measuring patterns of spatial *Prnp* variation, particularly in recently-infected populations. Further studies focused on understanding spatial patterns of genetic connectivity may help to resolve whether observed *Prnp* heterogeneity is reflective of stochastic genetic variation or is a consequence of spatial genetic structure.

Continued and long-term genotyping efforts in this region and others recently infected by chronic wasting disease may provide novel insights into the factors that influence prion-disease susceptibility in cervids and other species. Selection dynamics for prion diseases are complex and there is no consensus on the relative importance of balancing versus purifying selection for these or similar loci [43]. There also seems to be some indication from scrapie models of negative fitness effects of less susceptible alleles in naïve populations, which may affect patterns of selection [44]. In conjunction with previous work [26], minor allele homozygotes were rare in this region of recent infection, providing some support for this. Being able to track allele frequency change over the epizootic cycle, from emergence to endemism, may elucidate the selection dynamics of prion diseases. Additionally, continued study of this system may provide an important test regarding the relative influence of selection and demography on *Prnp* variability. While selection may occur, it will have to be strong enough to overcome migration rates from disparate populations with potentially different disease and genetic dynamics [21]. Finally, there is still some ambiguity regarding the importance of *Prnp* variability as a management tool [22,28]. Tracking the progression of the disease in tandem with changes in allele frequencies may provide managers with an opportunity to understand how important genetic susceptibility is to the health and persistence of infected populations.

## Methods

### Genetic sampling

7Tissue samples were collected from white-tailed deer in an area encompassing approximately 70,000 km^2^ in three Mid-Atlantic states between 2013 and 2017 (Figure 1). Current estimates indicate low chronic wasting disease prevalence rates (< 1.0%) in this region [32,33]. Therefore, it represents an ideal study area for determining patterns of susceptibility early in the epizootic cycle. We collected 728 tissue samples in coordination with regional disease surveillance programs that consisted of a small connective tissue biopsy, either from the ear or tongue of deer sampled from vehicle mortality or hunter harvest. Samples were grouped by county to assess differential patterns of disease susceptibility throughout the region. Geographic information was collected for spatial analyses and corresponded to the centroid of the county in which a sample was collected (Figure 1). Samples were then grouped into four subregions generally consistent with ecophysiographic regions for comparison. These four regions were separated by geographic boundaries predicted to influence population genetic structure. Sample sizes ranged from 78 (subregion 3) to 338 (subregion 2). Preliminary simulations indicated that sample sizes at both the county- and subregion-scale provide accurate estimates of genotype frequencies and are likely to be reflective of geographic patterns (Appendix B). Disease status for all deer was determined using immunohistochemical staining or immunoassay (ELISA) techniques by the collection agency. State agencies utilized either obex or retropharyngeal lymph tissue, or both, to test for the presence of infectious prion proteins.

### Prnp sequence analysis

Genomic DNA was extracted using the QIAGEN DNeasy blood and tissue extraction protocol (QIAGEN). We used a previously described primer set that encompasses the open reading frame of the *Prnp* gene to amplify the functional coding region via the polymerase chain reaction (PCR) [34]. The forward primer 223 (5ʹ – ACACCCTCTTTATTTTGCAG-3ʹ) complement sequence is located on intron two. The complementary sequence to reverse primer 224 (5ʹ – AGAAGATAATGAAAACAGGAAG-3ʹ) is located on exon three and is downstream of the stop codon. These primers are specific to the *Prnp* open-reading frame and do not amplify the *Prnp* pseudogene [34]. Polymerase chain reactions were carried out using the QIAGEN Multiplex PCR Master Mix. Total reaction volumes amounted to 10.00 µL: 5.00 µL 2x Master Mix, 1.00 µL 5x Q-Solution, 0.125 µL of 20 µM forward and reverse primers, 1.75 µL deionized H_2_O, 2.00 µL of 20 ng/µL DNA template. All PCRs were carried out using the following protocol: 95°C for 15 minutes, 35x cycles (95°C for 30 seconds, 60°C for 90 seconds, and 72°C for 60 seconds), with a final extension at 72°C for 10 minutes. Successful PCR amplifications were purified using an Exonuclease 1/Shrimp Alkaline Phosphatase cleaning procedure. Purification mixes consisted of 0.58 µL of Exonuclease 1 (20,000 units/mL concentration; New England BioLabs), 0.58 µL of Shrimp Alkaline Phosphatase (1,000 units/mL concentration; New England BioLabs), 8.84 µL deionized H_2_O, and 10 µL of PCR amplicon. The following thermocycler protocol was applied to Exonuclease 1/Shrimp Alkaline Phosphatase purification: 37°C for 30 minutes and 80°C for 15 minutes. Sanger sequencing was performed on an Applied Biosystems 3730 sequencing platform (Applied Biosystems) at the Penn State Genomics Core Facility.

All *Prnp* sequence reads were scored and analyzed using the CWDPRNP R package [45]. The CWDPRNP package was designed to perform automated sequence alignment and genotyping of cervids using electropherogram files from Applied Biosystems platforms. Four sequences were scored for each individual, which included primary and secondary sequence calls for forward and reverse reactions. Two sets of calls were recorded for each reaction to identify heterozygous loci. We used a threshold of 0.33 to score secondary peaks, which was adequate at detecting heterozygote sites while minimizing the spurious calling of spectral pull-up when compared to chromatograms. Forward and reverse complement sequences were aligned using the ClustalW algorithm [46]. All sequences were trimmed to a 771 base pair fragment encompassing the open reading frame previously described using this primer set (GenBank accession number AY275712) [34]. We filtered low-quality bases and sequence reads using Phred output files. Any sequence read that did not have > 75% of bases above a Phred score of 20 was removed. Because heterozygotes can be unintentionally filtered using Phred scores due the presence of two electrophoretic peaks of similar fluorescence, we did not filter individual loci by Phred scores. Instead, we filtered all bases where the forward and reverse complement sequences did not match in order to ensure read accuracy. Chromatograms were used to check that regions containing polymorphisms were of sufficient quality for scoring. We feel that this scoring regime would not substantially bias allele frequencies, as most previously reported polymorphisms occur outside of areas characterized by higher densities of low quality calls (the first and last 100 bases of the sequence) [26,29,38,37]. Finally, we obtained replicate sequences for 84 samples (11.5% of the total sample) to ensure quality and accuracy. We found no instances of incorrect calls at surveyed polymorphic sites.

### Analysis of sequence variation

We identified all variable sites within the *Prnp* gene and estimated genotype and allele frequencies of SNP loci. We translated nucleotide sequences into their respective amino acid sequence as disease-associated loci have been typically linked to nonsynonymous base substitutions and are more commonly discussed by their amino acid designation [25,26,28,37]. Genotype and allele frequencies were estimated for the entire sample and for each population. Only sampling locations with sample sizes ≥ 20 individuals, which equated to 15 sampling locations, were used in population-scale analyses to minimize the influence of small sample sizes on estimates of *Prnp* variability. Confirmation to Hardy-Weinberg expectations was evaluated for codons 95 and 96. Hardy-Weinberg tests were carried out in Genepop (version 4.6) [47,48] with samples stratified by sampling locality to account for potential substructure. Significance was assessed using a Holm-Bonferroni correction for multiple comparisons [49,50]. Because patterns of susceptibility may be additive [26], we also calculated the frequency of multi-locus genotypes for disease-associated loci.

We compared sample-wide genotype frequencies to those reported from previous studies to determine whether genotype frequencies differed significantly from those previously observed. Results from six previous studies were used for comparisons [25,26,28,29,35,36]. Two studies were conducted in the chronic wasting disease management zone of Wisconsin [25,28], so genotype frequencies were pooled into a single estimate. Because the frequency of minor allele (less susceptible) homozygotes was rare across all studies, we combined heterozygotes and minor allele homozygotes into a single category (less susceptible state). Pairwise Fisher’s exact tests were used to determine significance of comparisons following a Bonferroni correction for multiple comparisons (Bonferroni-corrected *P* value designated *P’*). Genotype frequencies were also compared among subregions using the same procedure to determine whether specific subpopulations existed that were more susceptible to chronic wasting disease invasion and establishment.

Prion gene variation was mapped throughout the region to visualize patterns of population susceptibility. Genotype frequencies were mapped to the centroid of each sampled county. Inverse-distance weighting interpolation was used to create a two-dimensional surface of the expected frequency of the most susceptible genotype across the region. The power parameter was set to three and seemed to appropriately balance weighting of more distant points (avoided concentric ‘bulls-eye’ pattern) and close-by points (appropriately smooths the surface and avoids tessellation).

## Figures and Tables

**Figure B1.**
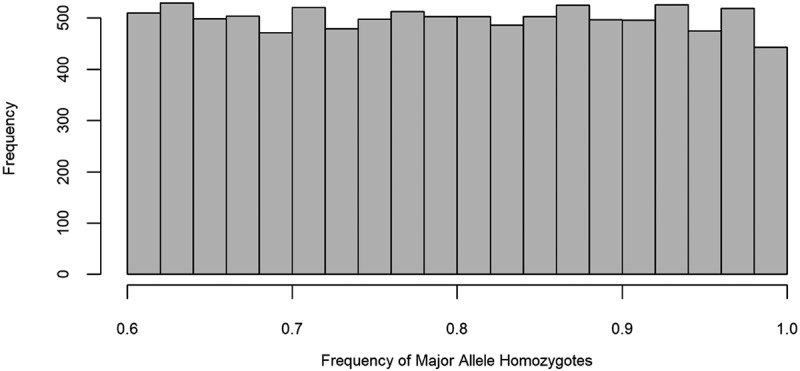
Frequency distribution of major allele homozygotes for simulated populations.

**Figure B2.**
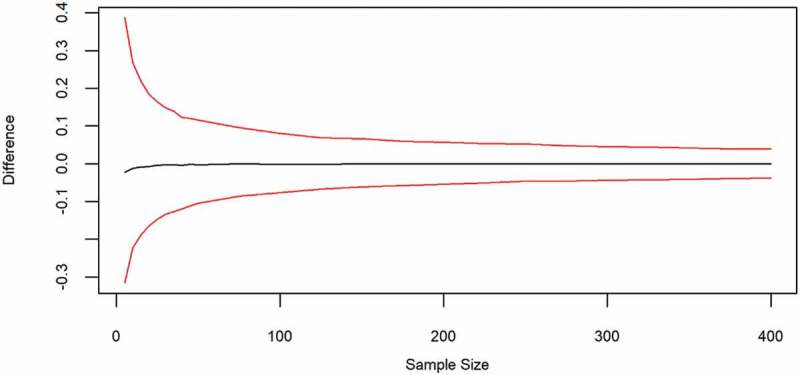
Median differences of genotype frequencies between simulated samples and true populations. Red lines indicate 0.025 and 0.975 quantiles of a distribution of 10,000 replicate simulations per sampling intensity level.

## Appendix A

*Prnp* genotypes of 27 white-tailed deer with chronic wasting disease. Samples were collected during routine state surveillance in the Mid-Atlantic region of the United States of America. Geographic location (state abbreviation, county, and subregion) are indicated. Deer sex is categorically listed (M = male, F = Female). Age classes are coded by months (30+ months = adult, 18 months = yearling, <6 months = fawn, NA = not available). *Prnp* loci are indicated by nucleotide number with amino acid residue indicated in parentheses.

**Table:**

			Age	60	110	153	285	286	324	367	378	438	499	555	676
Sample ID	Subregion	Sex	(Months)	(20)	(36)	(51)	(95)	(96)	(108)	(123)	(126)	(146)	(167)	(185)	(226)
MD_Allegany01	2	M	30+	C/C	G/G	C/C	A/A	G/G	A/A	G/G	G/G	T/C	A/A	T/C	C/C
PA_Bedford01	2	F	30+	C/C	G/G	T/C	A/A	G/A	A/A	G/G	G/G	C/C	A/A	T/C	C/C
PA_Bedford02	2	M	30+	C/C	G/G	C/C	A/A	G/G	A/A	G/G	G/G	C/C	A/A	C/C	C/C
PA_Bedford03	2	F	30+	C/C	G/G	C/C	A/A	G/G	A/A	G/G	G/G	C/C	A/A	T/C	C/C
PA_Bedford04	2	M	30+	C/C	G/G	C/C	A/A	G/G	A/A	G/G	G/G	C/C	A/A	C/C	C/C
PA_Bedford05	2	F	30+	C/C	G/G	C/C	A/A	G/G	A/A	G/G	G/G	T/T	A/A	C/C	C/C
PA_Bedford06	2	F	18	C/C	G/G	T/T	A/A	G/G	A/A	G/G	G/G	C/C	A/A	C/C	C/C
PA_Bedford07	2	M	18	C/C	G/G	T/C	A/A	G/G	A/A	G/G	G/G	C/C	A/A	T/C	C/C
PA_Bedford08	2	M	18	C/C	G/G	C/C	A/A	G/G	A/A	G/G	G/G	C/C	A/A	C/C	C/C
PA_Bedford09	2	M	30+	C/C	G/G	C/C	A/A	G/G	A/A	G/G	G/G	C/C	A/A	T/C	C/C
PA_Bedford10	2	M	30+	C/C	G/G	C/C	A/A	G/G	A/A	G/G	G/G	C/C	A/A	C/C	C/C
PA_Blair01	2	F	30+	C/C	G/G	C/C	A/A	G/A	A/A	G/G	G/G	C/C	A/A	T/T	C/C
PA_Blair02	2	M	30+	C/C	G/G	C/C	A/A	G/G	A/A	G/G	G/G	C/C	A/A	C/C	C/C
PA_Cambria01	3	F	30+	C/C	G/G	T/C	A/A	G/G	A/A	G/G	G/G	C/C	A/A	C/C	C/C
PA_Clearfield01	3	M	30+	C/C	G/G	C/C	A/A	G/G	A/A	G/G	G/G	C/C	A/A	C/C	C/C
PA_Clearfield02	3	M	30+	C/C	G/G	C/C	A/A	G/G	A/A	G/G	G/G	C/C	A/A	T/C	C/C
PA_Fulton01	2	M	30+	C/C	G/G	T/T	A/A	G/G	A/A	G/G	G/G	C/C	A/A	C/C	C/C
PA_Fulton02	2	F	30+	T/C	G/G	C/C	A/A	G/G	A/A	G/G	G/G	C/C	A/A	C/C	C/C
PA_Fulton03	2	F	30+	C/C	G/G	C/C	A/A	G/G	A/A	G/G	G/G	C/C	A/A	C/C	C/C
PA_Fulton04	2	M	18	C/C	G/G	C/C	A/A	G/G	A/A	G/G	G/G	C/C	A/A	T/C	C/C
PA_Fulton05	2	F	<6	C/C	G/G	C/C	A/A	G/G	A/A	G/G	G/G	T/C	A/A	T/C	C/C
PA_Fulton06	2	M	30+	C/C	G/G	T/C	A/A	G/G	A/A	G/G	G/G	T/C	A/A	C/C	C/C
PA_Fulton07	2	M	30+	C/C	G/G	T/C	A/A	G/G	A/A	G/G	G/G	C/C	A/A	C/C	C/C
PA_Fulton08	2	M	30+	C/C	G/G	C/C	A/A	G/G	A/A	G/G	G/G	T/C	A/A	C/C	C/C
VA_Shenandoah01	4	M	30+	C/C	G/G	C/C	A/A	G/G	A/A	G/G	G/G	T/C	A/A	C/C	C/C
VA_Frederick01	4	M	30+	C/C	G/G	C/C	A/A	G/G	A/A	G/G	G/G	C/C	A/A	T/T	C/C
VA_Frederick02	4	M	NA	C/C	G/G	C/C	A/A	G/G	A/A	G/G	G/G	C/C	A/A	T/C	C/C

## Appendix B

Simulations to determine effects of sample size on accuracy of estimated Prnp genotype frequencies.

### Simulation Methodology

We investigated the relationship between sample size and the accuracy of estimated Prnp genotype frequencies in program R. A total of 10,000 populations made up of 10,000 individuals each were simulated. Genotypes were binomially classified in a manner consistent with empirical data. The proportion of major allele homozygotes was varied for each population using a uniform distribution between 0.6 and 1.0 consistent with the possible frequencies of the most susceptible genotypes at disease-associated loci (Figure B1). All other individuals were assigned to a less susceptible state category, which represents a combination of heterozygote and minor allele homozygote genotypes.

We then calculated the difference between the simulated genotype frequencies and estimates derived by random subsampling from these simulated populations. Individuals were randomly drawn from each simulated population using sampling intensities between 0 and 400 individuals. Sample sizes were increased in increments of 5 between 0 and 50 and then by increments of 25 after that. We then calculated the difference between estimated frequencies of major allele homozygotes from the true genotypic frequency for each population. Difference estimates were pooled for each sampling intensity across all 10,000 populations. This distribution represented both the stochasticity involved in the sampling process and uncertainty regarding the true population genotype frequency. We used a threshold difference of ± 10% to evaluate the proportion of simulations that fall within these bounds.

**Table B1.** Percentage of simulations within ± 10% of true allele frequency stratified by sample size. Sample sizes below the 90% threshold are highlighted in gray. Sample sizes approximate to subregion sample sizes are colored in accordance with Figure 1 (orange = subregion 1, blue = subregion 2, green = subregion 3, yellow = subregion 4).

### Results

The median difference between sample and true genotype frequencies was < 1.0% for sample sizes as small as 15 individuals (Figure B2). Sample sizes of 40 or more have ≥ 90.4% of all simulations within ± 10% of true genotype frequencies (Table B1). At a county-scale, only 3 out of 15 counties have sample sizes below this level. Simulations with sampling intensities similar to that of subregion sample sizes contained > 97% of simulations within ±10% of true genotype frequencies (Table B1). These simulations indicate that our data are sufficiently accurate to describe geographic variability in Prnp frequencies at a county-and subregion-scale.
